# Prevalence and antimicrobial resistance of *Enterococcus* spp. isolated from animal feed in Japan

**DOI:** 10.3389/fvets.2023.1328552

**Published:** 2024-01-24

**Authors:** Yohei Yamagami, Miyuki Asao, Akiko Takahashi, Yoshiyasu Hashimoto, Noriko Okuyama, Eiko Arai, Wakana Arihara, Ryota Masui, Yoko Shimazaki

**Affiliations:** Department of Fertilizer and Feed Inspection, Food and Agricultural Materials Inspection Center, Saitama, Japan

**Keywords:** animal feed, *Enterococcus*, prevalence, antimicrobial resistance, resistance gene

## Abstract

The rising prevalence of antimicrobial resistance (AMR) of bacteria is a global health problem at the human, animal, and environmental interfaces, which necessitates the “One Health” approach. AMR of bacteria in animal feed are a potential cause of the prevalence in livestock; however, the role remains unclear. To date, there is limited research on AMR of bacteria in animal feed in Japan. In this study, a total of 57 complete feed samples and 275 feed ingredient samples were collected between 2018 and 2020. *Enterococcus* spp. were present in 82.5% of complete feed (47/57 samples), 76.5% of soybean meal (62/81), 49.6% of fish meal (55/111), 33.3% of poultry meal (22/66), and 47.1% of meat and bone meal (8/17) samples. Of 295 isolates, *E. faecium* (33.2% of total isolates) was the dominant *Enterococcus* spp., followed by *E. faecalis* (14.2%), *E. hirae* (6.4%), *E. durans* (2.7%), *E. casseliflavus* (2.4%), and *E. gallinarum* (1.0%). Of 134 isolates which were tested for antimicrobial susceptibility, resistance to kanamycin was the highest (26.1%), followed by erythromycin (24.6%), tetracycline (6.0%), lincomycin (2.2%), tylosin (1.5%), gentamicin (0.8%), and ciprofloxacin (0.8%). All *Enterococcus* spp. exhibited susceptibility to ampicillin, vancomycin, and chloramphenicol. Of 33 erythromycin-resistant isolates, only two showed a high minimum inhibitory concentration value (>128 μg/mL) and possessed *ermB*. These results revealed that overall resistance to antimicrobials is relatively low; however, animal feed is a source of *Enterococcus* spp. It is essential to elucidate the causative factors related to the prevalence of AMR in animal feed.

## 1 Introduction

The emergence and spread of antimicrobial resistance (AMR) of bacteria are a widely recognized global health threat (1). The concept of “One Health” is crucial to address this issue because humans, animals, food, and the environment are potential reservoirs of AMR of bacteria and resistance genes (2). Guidelines of OIE (3) and Codex Alimentarius (4) recommend the surveillance and monitoring of foodborne AMR of bacteria in livestock, animal feed, and so on. In Japan, foodborne AMR of bacteria in livestock have been monitored by the Japanese Veterinary Antimicrobial Resistance Monitoring System (JVARM) since 1999 (5). Despite their promotion of the appropriate use of antimicrobials, resistance to several antimicrobial agents in *Enterococcus* spp. isolated from healthy livestock exceeded 40%, according to JVARM (6).

As food-producing animals continue to make important contributions to our food supply, animal feed has become a critical component for producing safe food across the farm-to-table continuum. Bacteria in animal feed are a potential source that could influence the prevalence of AMR of bacteria in livestock (7). Animal feed includes feed ingredients, which are derived from animals and plants, as well as complete feed, which is a quantitative mixture of dietary ingredients to meet specific nutrient requirements. Water activity is low in many types of animal feed; however, the control of bacterial contamination is difficult because animal feed is not completely sterilized via heat treatment during feed production. *Enterococcus* is more common in animal feed than *Escherichia coli*, *Salmonella*, and *Campylobacter* (8–10). In our small-scale preliminary study, we analyzed the prevalence of *Enterococcus* spp. and *E. coli* in feed ingredients in Japan. We found *Enterococcus* spp. to be more prevalent than *E. coli*, which corroborated with the results of prior research (8, 9).

In previous studies, *Enterococcus* spp. isolated from animal feed in Portugal and the USA were resistant to several antimicrobial agents (8, 9, 11). These results revealed that AMR of *Enterococcus* spp. exists in a certain proportion of animal feed. Their AMR profile may differ for each country because many feed ingredients are manufactured from domestic ingredients. However, the AMR profile of animal feed in Japan has not been well-studied.

Monitoring and surveillance of foodborne AMR contributes to the food safety component of the “One Health” approach (4). In this study, we investigated the prevalence and AMR profile of *Enterococcus* spp., especially *E. faecalis* and *E. faecium*, in accordance with guidelines of OIE (3) and Codex Alimentarius (4), and as well as the presence of resistance genes in the predominant AMR of isolates. Moreover, we compared the resistance rates of isolates between animal feed and livestock to investigate whether animal feed is a potential cause of the prevalence of *Enterococcus* spp. in livestock.

## 2 Materials and methods

### 2.1 Sample collection

**Figure 1:**
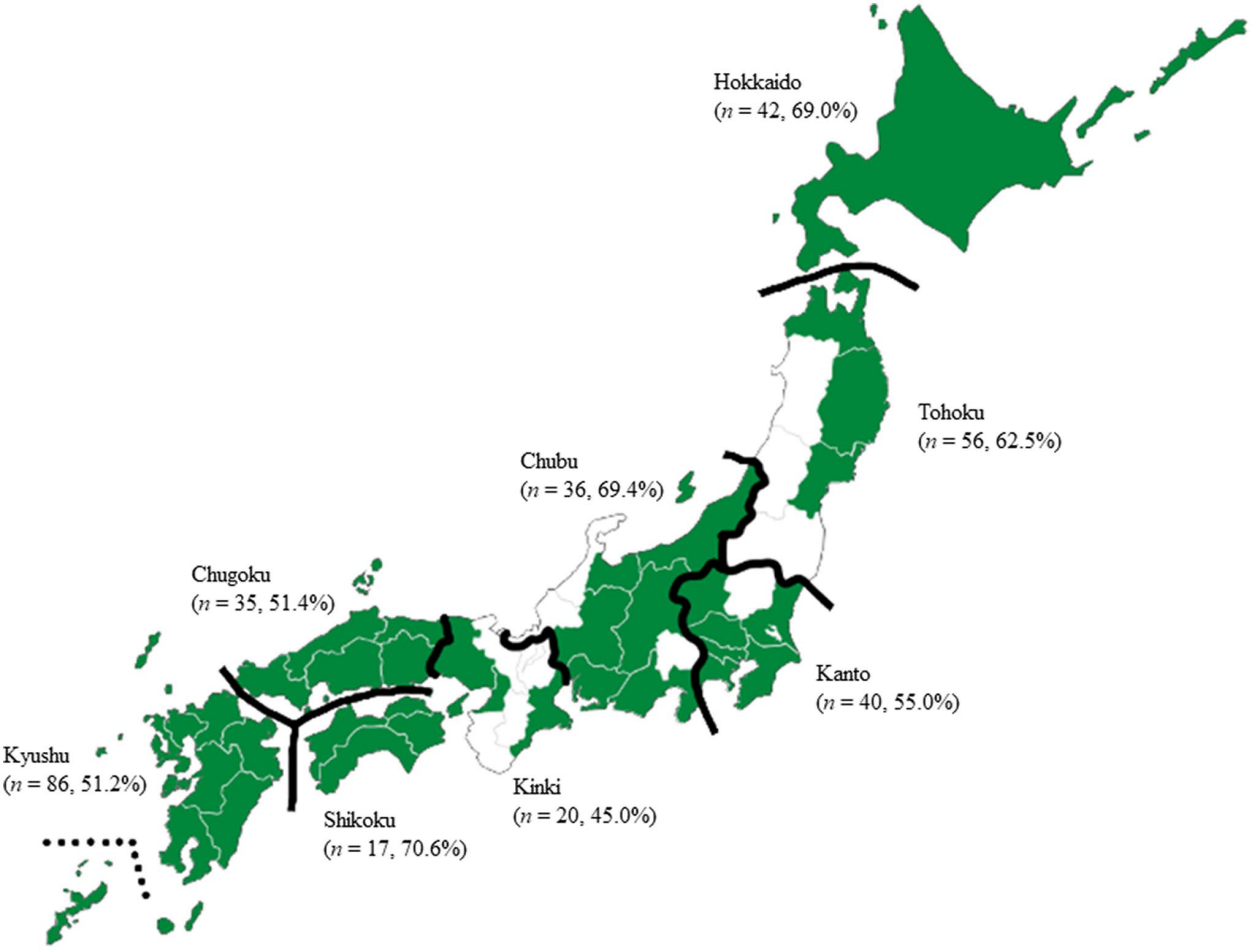
Map of Japan showing sampling locations of feed mill. All 47 prefectures of Japan were divided into eight regions (Hokkaido, Tohoku, Kanto, Chubu, Kinki, Chugoku, Shikoku, and Kyushu), and 180 feed mills from 36 prefectures were selected. Color indicated the prefectures sampling animal feed. *P*-values were determined using Fisher’s exact test. No significant differences were found in the prevalence of *Enterococcus* spp. in animal feed among the regions (*p* > 0.05). *n*, number of samples; %, prevalence of *Enterococcus* spp.

We collected samples of 57 complete animal feed (for 24 poultry, 13 swine, and 20 cattle), 81 soybean meal, 111 fish meal, 66 poultry meal, and 17 meat and bone meal, in which *Enterococcus* spp. were commonly found according to previous studies (8, 9, 11); however, we omitted complete feed samples that contained microbial feed supplements (also called probiotics) of live *E. faecalis* and *E. faecium*. To avoid bias, all 47 prefectures of Japan were divided into eight regions (Hokkaido, Tohoku, Kanto, Chubu, Kinki, Chugoku, Shikoku, and Kyushu) (Figure 1), and 180 feed mills from 36 prefectures were selected. Animal feed samples (250 g) were collected from paper sacks (except for poultry meal and meat and bone meal), flexible intermediate bulk containers, or feed trucks in the feed mills between 2018 and 2020. A total of 332 animal feed samples (57 complete feed samples and 275 feed ingredients) were placed in sterile bags and kept refrigerated until tested.

### 2.2 Isolation and identification of bacteria

Twenty-five grams of each sample was mixed thoroughly with AC Broth Base (Nissui Pharmaceutical, Tokyo, Japan) and incubated at 37°C for 18–48 h. One loop of the enriched sample was inoculated on an Enterococcosel agar plate (Becton, Dickinson and Co., Sparks, MD, USA, and Kyokuto Pharmaceutical Industrial, Tokyo, Japan) and incubated at 37°C for 18–72 h. One or two predominant colonies per sample, presumptively isolated as enterococci through colony morphology (i.e., dark brown halo), were inoculated on brain-heart infusion agar plates (Becton, Dickinson and Co.) and incubated at 37°C for 18–24 h. Evaluation of Gram-staining, growth in heart infusion broth (Becton, Dickinson and Co.) supplemented with 6.5% NaCl and at 45°C, pigmentation, and motility in motility test medium (Becton, Dickinson and Co.) with triphenyl-tetrazolium chloride indicator was performed. Suspected *Enterococcus* spp. isolates were identified to the genus and species levels with an API rapid ID 32 STREP kit (bioMérieux, Lyon, France) and multiplex polymerase chain reaction (PCR) assay (further details regarding the identification process of bacteria are provided in Supplementary File 1). Amplification of species-specific genes was performed to identify the following species: *E. faecalis*, *E. faecium*, *E. casseliflavus*, *E. durans*, *E. gallinarum*, and *E. hirae*. Template DNA was extracted from colonies using the InstaGene^™^ Matrix (Bio-Rad Laboratories, Hercules, CA, USA), according to the manufacturer’s instructions. PCR was performed as previously described (12) with some modifications (see Supplementary File 2).

### 2.3 Antimicrobial susceptibility testing

Of the 295 isolates, one isolate per species was selected from each sample, after which 134 isolates were subjected to antimicrobial susceptibility testing. The test was performed by the broth microdilution method using an Eiken frozen plate (Eiken Chemical, Tokyo, Japan), according to the manufacturer’s instructions. Two-fold dilution of antimicrobial agents were prepared in a 96-well U-shaped microplates, and final concentration ranges for 10 antimicrobial agents were as follows: ampicillin, 0.12–128 μg/mL; vancomycin, 0.12–128 μg/mL; tetracycline, 0.12–64 μg/mL; erythromycin, 0.12–128 μg/mL; tylosin, 0.12–128 μg/mL; lincomycin, 0.25–512 μg/mL; gentamicin, 0.12–256 μg/mL; kanamycin, 0.25–512 μg/mL; chloramphenicol, 0.25–512 μg/mL; and ciprofloxacin, 0.12–128 μg/mL. Inoculum preparation, inoculation, incubation, and determining microdilution end points were performed according to the guidelines of the Clinical and Laboratory Standards Institute (CLSI) (13). The CLSI breakpoints were used for ampicillin, vancomycin, tetracycline, erythromycin, chloramphenicol, and ciprofloxacin (14). Microbiologically determined JVARM breakpoints were used for the other antimicrobial agents (6) because their breakpoints are not established by CLSI (14). *E. faecalis* ATCC 29212 was used as a quality control.

### 2.4 Detection of resistance genes

The macrolide- and aminoglycoside-resistant isolates were subjected to PCR assay to detect the presence of resistance genes associated with erythromycin (*ermA*, *ermB*, and *mefA/E*) (15) and aminoglycosides (*aac(6′)-aph(2″)*, and *aph(3′)-IIIa*) (16). Template DNA isolation and PCR were performed as previously described (17).

### 2.5 Resistance rates of isolates in livestock

To determine the resistance rates of isolates in livestock, we used JVARM data on *Enterococcus* spp. isolates recovered from fecal samples collected in a slaughterhouse between 2018 and 2019 (6). The mean resistance rates of isolates from poultry, swine, and cattle were calculated and compared to those of isolates from animal feed.

### 2.6 Statistical analysis

A two-tailed Fisher’s exact test in R version 4.3.1 (18) was used to compare the prevalence of *Enterococcus* spp. among regions of Japan as well as among animal feed samples, and the resistance rates of isolates in animal feed with those in livestock. Statistical significance was set as *p* < 0.05.

## 3 Results

### 3.1 Prevalence of *Enterococcus* spp.

The prevalence of *Enterococcus* spp. in animal feed samples in regions of Japan, namely Hokkaido, Tohoku, Kanto, Chubu, Kinki, Chugoku, Shikoku, and Kyushu was 69.0, 62.5, 55.0, 69.4, 45.0, 51.4, 70.6, and 51.2%, respectively (Figure 1). No significant differences among regions were found (*p* > 0.05). The prevalence of *Enterococcus* spp. in animal feed samples is shown in Table 1. The prevalence in complete feed was higher than that in feed ingredient (*p* < 0.05). In feed ingredients, the prevalence in soybean meal was higher than that in fish meal, poultry meal, and meat and bone meal (*p* < 0.05). In animal-derived feed, the prevalence in fish meal was significantly higher than that in poultry meal (*p* < 0.05). Of 295 isolates, *E. faecium* (33.2% of total isolates) was the dominant *Enterococcus* spp., followed by *E. faecalis* (14.2%), *E. hirae* (6.4%), *E. durans* (2.7%), *E. casseliflavus* (2.4%), and *E. gallinarum* (1.0%). The prevalence of other *Enterococcus* spp. was 40.0%. *E. faecium* was the predominant species in complete feed, soybean meal, fish meal, and meat and bone meal, whereas *E. faecalis* was the predominant species in poultry meal.

**Table 1:** Prevalence of *Enterococcus* spp. in complete feed and feed ingredient samples.

Animal feed type	No. of samples	No. (%) of positive samples		No. of isolates	*E. faecium*		*E. faecalis*		*E. hirae*		*E. durans*		*E. casseliflavus*		*E. gallinarum*		Other	
Complete feed	57	47	(82.5)^A^	72	35		5		3		3		0		0		26	
Poultry feed	24	18	(75.0)^a^	18	8		2		0		0		0		0		8	
Swine feed	13	11	(84.6)^a^	22	12		2		3		0		0		0		5	
Cattle feed	20	18	(90.0)^a^	32	15		1		0		3		0		0		13	
Feed ingredients	275	147	(53.5)^B^	223	63		37		16		5		7		3		92	
Soybean meal	81	62	(76.5)^a^	97	29		3		8		2		4		2		49	
Fish meal	111	55	(49.6)^b^	82	26		16		2		2		3		0		33	
Poultry meal	66	22	(33.3)^c^	31	3		15		5		1		0		1		6	
Meat and bone meal	17	8	(47.1)^bc^	13	5		3		1		0		0		0		4	
Total (%)	332	194	(58.4)	295	98	(33.2)	42	(14.2)	19	(6.4)	8	(2.7)	7	(2.4)	3	(1.0)	118	(40.0)

*P*-values were determined using Fisher’s exact test. Percentages followed by different uppercase letters (A or B) were significantly different (*p* < 0.05) for the prevalence between complete feed and feed ingredients. Percentages followed by different lowercase letters (a, b, or c, but not bc) were significantly different (*p* < 0.05) for the prevalence within complete feed or feed ingredients.

### 3.2 Antimicrobial susceptibility

Resistance rates of isolates in each animal feed sample are shown in Table 2. In antimicrobial susceptibility testing, erythromycin resistance rates were 26.3–33.3%, except for poultry meal (0.0%). Kanamycin resistance rates were 13.3–37.1%, except for meat and bone meal (0.0%). Other resistance rates were below approximately 10%. All *Enterococcus* spp. exhibited susceptibility to ampicillin, vancomycin, and chloramphenicol.

**Table 2:** Resistance rates of enterococcal isolates in complete feed and feed ingredient samples.

		No. (%) of resistant isolates													
Animal feed type	No. of isolates	Tetracycline		Erythromycin		Tylosin		Lincomycin		Gentamicin		Kanamycin		Ciprofloxacin	
Complete feed	35	2	(5.7)	9	(25.7)	1	(2.9)	1	(2.9)	0		13	(37.1)	1	(2.9)
Feed ingredients	99	6	(6.1)	24	(24.2)	1	(1.0)	2	(2.0)	1	(1.0)	22	(22.2)	0	
Soybean meal	40	2	(5.0)	12	(30.0)	1	(2.5)	1	(2.5)	0		13	(32.5)	0	
Fish meal	38	2	(5.3)	10	(26.3)	0		1	(2.6)	0		7	(18.4)	0	
Poultry meal	15	2	(13.3)	0		0		0		1	(6.7)	2	(13.3)	0	
Meat and bone meal	6	0		2	(33.3)	0		0		0		0		0	
Total (%)	134	8	(6.0)	33	(24.6)	2	(1.5)	3	(2.2)	1	(0.8)	35	(26.1)	1	(0.8)

All *Enterococcus* spp. exhibited susceptibility to ampicillin, vancomycin, and chloramphenicol.

The minimum inhibitory concentration (MIC) of isolates from animal feed samples is shown in Table 3. For all *Enterococcus* spp. isolates, resistance to kanamycin was the highest (26.1%), followed by erythromycin (24.6%), tetracycline (6.0%), lincomycin (2.2%), tylosin (1.5%), gentamicin (0.8%), and ciprofloxacin (0.8%). The major kanamycin- and erythromycin-resistant isolates were *E. faecium*. All *E. durans*, *E. gallinarum*, and *E. hirae* isolates were susceptible to all antimicrobial agents.

**Table 3:** MIC of enterococcal isolates in complete feed and feed ingredient samples, and resistant rates in animal feed and livestock.

Antimicrobial agent	Breakpoint (μg/mL)	Species	No. of isolates	Range (μg/mL)	MIC_50_ (μg/mL)	MIC_90_ (μg/mL)	No. of resistant isolates	Resistant rate (%)^c^	
								In animal feed	In livestock
Ampicillin	16^a^	All *Enterococcus* spp.	134	≤0.12~2	1	2	0	0.0	0.1
		*E. faecalis*	31	0.5~2	1	1	0	0.0	0.0
		*E. faecium*	75	≤0.12~2	2	2	0	0.0	0.0
Vancomycin	32^a^	All *Enterococcus* spp.	134	≤0.12~8	0.5	4	0	0.0	0.0
		*E. faecalis*	31	0.5~4	1	2	0	0.0	0.0
		*E. faecium*	75	0.25~4	0.5	4	0	0.0	0.0
Tetracycline	16^a^	All *Enterococcus* spp.	134	≤0.12~>64	0.25	0.5	8	6.0	46.9*
		*E. faecalis*	31	0.25~>64	0.5	64	4	12.9	52.0*
		*E. faecium*	75	≤0.12~>64	0.25	0.5	3	4.0	27.9*
Erythromycin	8^a^	All *Enterococcus* spp.	134	≤0.12~>128	2	8	33	24.6	21.2
		*E. faecalis*	31	≤0.12~4	2	2	0	0.0	36.1*
		*E. faecium*	75	≤0.12~>128	4	8	32	42.7	11.4*
Tylosin	64^b^	All *Enterococcus* spp.	134	0.5~>128	4	8	2	1.5	20.5*
		*E. faecalis*	31	2~8	2	2	0	0.0	36.4*
		*E. faecium*	75	1~>128	4	8	2	2.7	8.1
Lincomycin	128^b^	All *Enterococcus* spp.	134	≤0.25~>512	16	32	3	2.2	26.8*
		*E. faecalis*	31	0.5~256	32	32	1	3.2	37.5*
		*E. faecium*	75	0.5~>512	16	32	2	2.7	8.1
Gentamicin	32^b^	All *Enterococcus* spp.	134	0.25~>256	8	16	1	0.8	11.3*
		*E. faecalis*	31	4~>256	16	16	1	3.2	22.8*
		*E. faecium*	75	2~16	4	8	0	0.0	8.3*
Kanamycin	128^b^	All *Enterococcus* spp.	134	2~>512	64	128	35	26.1	31.6
		*E. faecalis*	31	32~>512	64	64	2	6.5	43.2*
		*E. faecium*	75	32~512	64	128	33	44.0	46.0
Chloramphenicol	32^a^	All *Enterococcus* spp.	134	2~16	4	8	0	0.0	8.3*
		*E. faecalis*	31	4~8	8	8	0	0.0	21.1*
		*E. faecium*	75	2~16	4	8	0	0.0	6.4
Ciprofloxacin	4^a^	All *Enterococcus* spp.	134	≤0.12~4	0.5	1	1	0.8	7.8*
		*E. faecalis*	31	0.25~2	1	1	0	0.0	2.8
		*E. faecium*	75	0.25~4	0.5	1	1	1.3	10.5

^a^ Established by the Clinical and Laboratory Standards Institute (14).

^b^ Determined microbiologically (midpoint of a bimodal MIC distribution) by the Japanese Veterinary Antimicrobial Resistance Monitoring System (6).

^c^ All *Enterococcus* spp. (*n* = 861), *E. faecalis* (*n* = 228), and *E. faecium* (*n* = 20) in livestock. *P*-values were determined using Fisher’s exact test. *p < 0.05.

MIC_50_, minimum inhibitory concentration at which 50% of isolates are inhibited; MIC_90_, MIC at which 90% of isolates are inhibited.

### 3.3 Resistance genes

In the 33 erythromycin-resistant isolates, *ermB* was detected in two *E. faecium* isolates (6.1%, MIC of erythromycin >128 μg/mL) from swine feed and soybean meal. In contrast, *ermA* and *mefA/E* were not detected in any of the isolates.

In the 36 aminoglycoside-resistant isolates, *aac(6′)-aph(2″)* was detected in one *E. faecalis* isolate (2.8%, MIC of gentamicin >256 μg/mL) from poultry meal, whereas *aph(3′)-IIIa* was detected in another *E. faecalis* isolate (2.8%, MIC of kanamycin >512 μg/mL) from poultry meal.

### 3.4 Comparison of resistance rates of *Enterococcus* spp. between animal feed and livestock

The prevalence of *Enterococcus* spp. differed between animal feed and livestock. *E. hirae* was a major *Enterococcus* spp. in livestock (19) but a minor one in animal feed. Hence, we compared the resistance rates of *E. faecium* and *E. faecalis*, which were commonly isolated from both sources, because of considerable differences in antimicrobial susceptibility between them. The results are presented in Table 3. *E. faecium* in animal feed had significantly lower resistance rates to tetracycline and gentamicin than that in livestock (*p* < 0.05). In contrast, *E. faecium* in animal feed had significantly higher resistance rates to erythromycin than that in livestock (*p* < 0.05). Further, *E. faecalis* in animal feed had significantly lower resistance rates to tetracycline, erythromycin, tylosin, lincomycin, gentamicin, kanamycin, and chloramphenicol than that in livestock (*p* < 0.05). Notably, the overall resistance rates for both *E. faecium* and *E. faecalis* were lower in animal feed than in livestock. Lastly, all *Enterococcus* spp. obtained from animal feed had significantly lower resistance rates to tetracycline, tylosin, lincomycin, gentamicin, chloramphenicol, and ciprofloxacin than those obtained from livestock (*p* < 0.05).

## 4 Discussion

The prevalence of *Enterococcus* spp. in complete feed was higher than that in feed ingredient (*p* < 0.05). These results were similar to those reported in USA (83.3–86.2% in complete feed for poultry, swine, and cattle, higher than 54.0% in overall feed ingredients) (8). Other feed ingredients, such as blood meal, feather meal, alfalfa meal, oilseed byproducts, and corn byproducts, which are often mixed in complete feed, are also frequently contaminated with *Enterococcus* spp. (9). This mixing of *Enterococcus* spp.-contaminated feed ingredients may have impacted the results. Additionally, the prevalence of *Enterococcus* spp. in soybean meal was higher than that in fish meal, poultry meal, and meat and bone meal (*p* < 0.05). These results were similar to those reported in USA (100% in soybean meal and fish meal, higher than 88.2% in poultry meal, and 86.1% in meat and bone meal) (9). The manufacturing process of feed ingredients differs between plant- and animal-derived feed. Soybean meal is extracted from soybean using solvents such as hexane. In contrast, fish meal, poultry meal, and meat and bone meal are heat-treated at 100°C or more for a long time. The different abundances of *Enterococcus* spp. between plant- and animal-derived feed may be explained by the effect of heat treatment.

The tetracycline resistance rate was 6.0% (5.7% in complete feed and 6.1% in feed ingredients) in this study. This value was lower than that reported in Portugal (69.1% in poultry feed and 18.0% in feed ingredients) (11) and the USA (28.9% in complete feed) (8). Glycopeptide and fluoroquinolone antibiotics are critically important medicines for humans and animals. This study revealed low resistance rates for vancomycin and ciprofloxacin, which corroborated with the studies in Portugal (11) and USA (8, 9) (both below 10%).

In this study, erythromycin-resistant isolates were common in animal feed [24.6%, higher than the 7.1% observed in the USA (8)], soybean meal [30.0% vs. 27.8% in Portugal (11)], fish meal (26.3% vs. 22.2% in Portugal), and meat and bone meal. Erythromycin has been widely used in veterinary and human medicine. Macrolide antibiotics are important antimicrobial agents in human health and are used to treat community-acquired pneumonia, Legionnaires’ disease, and respiratory infections such as pertussis. Further, macrolide antibiotics are used to treat various diseases including pneumonia, bronchitis, and laryngitis in livestock. Erythromycin-resistant *Enterococcus* spp. in animal feed need to be closely monitored as they may enter the food chain.

Further, most MIC values of erythromycin-resistant isolates were close to the breakpoint (8 μg/mL), and these isolates did not possess *ermA*, *ermB*, or *mefA/E*. In contrast, erythromycin-resistant *E. faecium* that showed a high MIC value (>128 μg/mL) possessed *ermB*. MIC values of erythromycin-resistant *Enterococcus* spp. possessing *ermB* were > 128 μg/mL (20), and those isolated from humans, animals, and food possessed *ermB* > 80% (21, 22). The results revealed that the incidence of *ermB* in isolates from animal feed and from humans, animals, and food differed markedly.

Our findings revealed that the overall resistance rates for both *E. faecium* and *E. faecalis* were lower in animal feed than in livestock. Moreover, there were no chloramphenicol-resistant enterococcal isolates or erythromycin- and tylosin-resistant *E. faecalis* isolates in livestock (6). Ge et al. compared the resistance rates of *Enterococcus* spp. among animal feed, retail meat, and animals in the USA (8). The report suggested that animal feed was unlikely to significantly contribute to the resistance rates in retail products because the overall resistance was much lower in animal feed (8). In this study, the low prevalence of AMR of *Enterococcus* spp. in animal feed compared to that in livestock corroborates with this report. However, comparative gene analysis is needed to reveal the relationships among the isolates.

In conclusion, this study is the first nationwide investigation which revealed the prevalence and AMR profile of *Enterococcus* spp. in animal feed, as well as the presence of resistance genes in the predominant AMR of isolates in Japan. *Enterococcus* spp. was highly prevalent in a variety of domestic animal feed. *E. faecium* was the predominant species in complete feed, soybean meal, fish meal, and meat and bone meal, whereas *E. faecalis* was the predominant species in poultry meal. These were susceptible to many antimicrobial agents, including vancomycin and ciprofloxacin. Erythromycin-resistant *Enterococcus* spp. was commonly found; however, most isolates showed low MIC values and did not possess erythromycin-resistant genes, which have been consistently detected in enterococcal isolates from humans, animals, and food, according to previous studies. These major results revealed that overall resistance to antimicrobials is relatively low; however, animal feed is a source of *Enterococcus* spp. These suggest that animal feed plays little role in introducing AMR of bacteria into livestock, whereas it is essential to elucidate the causative factors related to the prevalence of AMR in animal feed. Additionally, there is a need for continued monitoring, especially of erythromycin-resistant *Enterococcus* spp. in animal feed, and comparative gene analysis to reveal the relationships between the AMR of *Enterococcus* spp. in animal feed and livestock.

## Data availability statement

The raw data supporting the conclusions of this article will be made available by the authors, without undue reservation.

## Author contributions

YY: Conceptualization, Data curation, Formal Analysis, Investigation, Methodology, Visualization, Writing – original draft, Writing – review & editing. MA: Conceptualization, Data curation, Investigation, Methodology, Writing – review & editing. AT: Data curation, Investigation, Writing – review & editing. YH: Conceptualization, Project administration, Writing – review & editing. NO: Investigation, Writing – review & editing. EA: Investigation, Writing – review & editing. WA: Investigation, Writing – review & editing. RM: Investigation, Writing – review & editing. YS: Visualization, Writing – review & editing.
