# Supplementary material for: Prevalence and antimicrobial resistance of Enterococcus spp. isolated from animal feed in Japan
Source: Front Vet Sci. 2024 Jan 24;10:1328552. doi: 10.3389/fvets.2023.1328552 (PMC10847256; doi:10.3389/fvets.2023.1328552)
Supplement: Supplementary file 1 [file Data_Sheet_1.docx]

# Identification process of bacteria

The identification process of bacteria was conducted as follows: Suspected *Enterococcus* spp. isolates were identified to the genus and species levels using an API rapid ID 32 STREP kit (bioMérieux, Lyon, France) and multiplex polymerase chain reaction (PCR) assay. 1) If suspected *Enterococcus* spp. isolates were detected at the species level using both methods, they were determined to be *Enterococcus* spp.; but 2) if suspected *Enterococcus* spp. isolates were detected at the species level using only one method, they were determined to be *Enterococcus* spp. with that particular method. There was no difference between the isolates of different species detected with an API rapid ID 32 STREP kit and PCR assay. One of the challenges faced during this procedure was that some suspected *Enterococcus* spp. isolates tested with an API rapid ID 32 STREP kit were identified to the genus level.
