# Supplementary material for: Prevalence and antimicrobial resistance of Enterococcus spp. isolated from animal feed in Japan
Source: Front Vet Sci. 2024 Jan 24;10:1328552. doi: 10.3389/fvets.2023.1328552 (PMC10847256; doi:10.3389/fvets.2023.1328552)
Supplement: Supplementary file 2 [file Data_Sheet_2.docx]

# Polymerase chain reaction (PCR)

The procedure for the original PCR assay (12) is as follows: Seven PCR master mixes consisting of different primer sets were prepared. Group 1 was *E. durans*, *E. faecalis*, *E. faecium*, and *E. malodoratus*; group 2 was *E. casseliflavus*, *E. gallinarum*, and *E. solitarius*; group 3 was *E. dispar*, *E. pseudoavium*, and *E. saccharolyticus*; group 4 was *E. flavescens*, *E. mundtii*, and *E. sulfureus*; group 5 was *E. avium*, *E. columbae*, and *E. seriolicida*; group 6 was *E. cecorum*, *E. hirae*, and *E. raffinosus*; and group 7 was *E. asini*, *E. gilvus*, *E. pallens*, and *E. porcinus/villorum*. The base master mix consisted of 3 mM MgCl_2_ (with Ficoll and tartrazine; Idaho Technology, Salt Lake City, UT, USA), 0.2 mM deoxynucleoside triphosphate mix (Roche, Indianapolis, IN, USA), 16 mM (10×) NH_4_, 3.5 U of Expand high-fidelity PCR system (Roche), and 1.25 µL of each genus primer (16 µM). With the exception of *E. faecalis*, *E. malodoratus*, *E. gallinarum*, *E. saccharolyticus*, and *E. dispar*, 1.25 µL of each species primer (16 µM) were added to the base mix. Further, 2.5 µL each of the primers of *E. faecalis*, *E. malodoratus*, *E. gallinarum*, *E. saccharolyticus*, and *E. dispar* were used. PCR was performed with a final volume of 22.5 µL consisting of 20 µL of master mix and 2.5 µL of whole-cell template. Following an initial denaturation at 95 °C for 4 min, the products were amplified with 30 cycles of denaturation at 95 °C for 30 s, annealed at 55 °C (groups 1, 2, 5, and 6) or 60 °C (groups 3, 4, and 7) for 1 min, and elongated at 72 °C for 1 min. Amplification was followed by a final extension at 72 °C for 7 min.

We performed PCR as previously described (12) and modified the grouping of *Enterococcus* spp., PCR reagents, concentration of each primer, final volume, and PCR cycle (for only *E. casseliflavus*). The details are as follows: Four PCR master mixes consisting of different primer sets were prepared. Group A was *E. durans*, *E. faecalis*, and *E. faecium*; group B was *E. casseliflavus*; group C was *E. gallinarum*; and group D was *E. hirae*. The base master mix consisted of 1.5 mM MgCl_2_, 0.2 mM deoxynucleoside triphosphate mix, 1× gold buffer, 2.0 U of AmpliTaq Gold (all PCR reagents were purchased from Thermo Fisher Scientific, Waltham, MA, USA), and 0.8 µM of each primer. PCR was performed with a final volume of 25.0 µL consisting of 22.5 µL of master mix and 2.5 µL of whole-cell template. Following an initial denaturation at 95 °C for 4 min (groups A, C, and D) or 10 min (group B), the products were amplified with 30 cycles of denaturation at 95 °C for 30 s, annealed at 55 °C (groups A, C, and D) or 45 °C (group B) for 1 min, and elongated at 72 °C for 1 min. Amplification was followed by a final extension at 72 °C for 7 min. *E. durans* (ATCC 6056), *E. faecalis* (ATCC 29212), *E. faecium* (ATCC 6057), *E. casseliflavus* (ATCC 700327), *E. gallinarum* (ATCC 49753), and *E. hirae* (ATCC 8043) were obtained from the American Type Culture Collection and used as positive controls. We confirmed that all positive controls were amplified in all assays.
